# Electrophysiological properties of vestibular hair cells isolated from human crista

**DOI:** 10.3389/fneur.2024.1501914

**Published:** 2025-01-22

**Authors:** Nesrien Mohamed, Mohammad Al-Amin, Frances L. Meredith, Olivia Kalmanson, Anna Dondzillo, Stephen Cass, Samuel Gubbels, Katherine J. Rennie

**Affiliations:** Department of Otolaryngology-Head & Neck Surgery, University of Colorado School of Medicine, Aurora, CO, United States

**Keywords:** vestibular hair cell, semicircular canal, K^+^ current, cGMP, vestibular schwannoma

## Abstract

**Introduction:**

The vast majority of cellular studies on mammalian vestibular hair cells have been carried out in rodent models due in part to the inaccessibility of human inner ear organs and reports describing electrophysiological recordings from human inner ear sensory hair cells are scarce. Here, we obtained freshly harvested vestibular neuroepithelia from adult translabyrinthine surgical patients to obtain electrophysiological recordings from human hair cells.

**Methods:**

Whole cell patch clamp recordings were performed on hair cells mechanically isolated from human cristae to characterize voltage-dependent and pharmacological properties of membrane currents. Hair cells were classified as type I or type II according to morphological characteristics and/or their electrophysiological properties.

**Results:**

Type I hair cells exhibited low voltage-activated K^+^ currents (IKLV) at membrane potentials around the mean resting membrane potential (-63 mV) and large slowly activating outward K^+^ currents in response to depolarizing voltage steps. Recordings from type II hair cells revealed delayed rectifier type outward K^+^ currents that activated above the average resting potential of -55 mV and often showed some inactivation at more depolarized potentials. Perfusion with the K^+^ channel blocker 4-aminopyridine (1 mM) substantially reduced outward current in both hair cell types. Additionally, extracellular application of 8-bromo-cGMP inhibited IKLV in human crista type I hair cells suggesting modulation via a nitric oxide/cGMP mechanism. A slow hyperpolarization-activated current (Ih) was observed in some hair cells in response to membrane hyperpolarization below -100 mV.

**Discussion:**

In summary, whole cell recordings from isolated human hair cells revealed ionic currents that strongly resemble mature current phenotypes previously described in hair cells from rodent vestibular epithelia. Rapid access to surgically obtained adult human vestibular neuroepithelia allows translational studies crucial for improved understanding of human peripheral vestibular function.

## 1 Introduction

Hair cells are the primary sensory cells within the inner ear and allow the conversion of mechanical signals into electrical receptor potentials and conveyance of information to the brainstem via afferents in the VIIIth cranial nerve. Precise processing of mechanosensory information enables hearing and balance function. Loss of hair cells can occur due to genetic causes, ototoxicity, noise exposure or aging resulting in hearing loss and vestibular dysfunction. In vestibular epithelia a considerable body of work from birds, reptiles and rodents has paved the way to a clearer understanding of hair cell properties and their roles in vestibular function in amniote species. However, only a few studies have been carried out on inner ear neuroepithelia from humans. Electively terminated fetal tissue (1, 2), cadaveric tissue and surgically obtained tissue (3–7) have been used to study human vestibular hair cell characteristics. Fresh vestibular tissue can be obtained when the inner ear is intentionally removed by surgical approaches and gives an opportunity to study otherwise discarded inner ear neuroepithelia. Pathology warranting this kind of surgery includes intracranial tumors like vestibular schwannomas, granulomas or meningiomas and refractory Meniere’s disease. We created a protocol at our institution to collect inner ear neuroepithelia for research studies and describe here a procedure for isolating human vestibular hair cells for electrophysiological and pharmacological studies.

In the vestibular organs of amniotes two types of hair cells were first identified based on morphological characteristics (8). Type I hair cells are amphora-shaped and make afferent synapses with large cup-shaped afferent dendrites called calyx terminals. Type II hair cells are roughly cylindrical with basolateral protuberances and contact afferent bouton synapses. Efferent neurons terminate on type II hair cells and on calyx terminals. In addition to anatomical differences, mature cell types also differ in their ionic conductances. Type I hair cells from rodents, birds and reptiles express low-voltage (called I_K,L_ or I_KLV_) and high-voltage (I_KHV_) activated potassium currents reviewed in Eatock and Songer (9) and Meredith and Rennie (10). Type II hair cells lack I_KLV_ but have I_KHV_ consisting of sustained delayed rectifier current as well as inactivating A-type K^+^ currents (11, 12). Both hair cell types can express hyperpolarization-activated currents (I_h_) at strongly negative potentials (13, 14). These clear-cut differences in mature hair cell populations have consequences for how the different hair cell types process sensory information in rodent vestibular neuroepithelia (11, 14, 15).

In avian vestibular epithelia hair cells can be routinely replaced following loss and non-sensory supporting cells undergo conversion to form functional new hair cells (16–19). Vestibular hair cell regeneration can occur following bilateral ablation of hair cells in mouse vestibular epithelia, but vestibular function does not fully recover (20–22). In mouse utricular epithelia, regenerated hair cells showed morphological and electrophysiological properties consistent with immature type II hair cells and no type I hair cell phenotypes were found (23). Recent data suggest hair cell regeneration also occurs in adult human vestibular epithelia promoting the prospect of hair cell replacement as a therapeutic approach for correction of balance disorders (5–7). Interestingly, transcriptomes for human hair cells and supporting cells show only a mild correlation with those in mouse (7).

In the current study vestibular end organs were extracted from adult patients undergoing surgeries for lateral skull base tumors across a broad age range. We found that biophysically viable hair cells can be mechanically dissociated following surgical extraction of cristae from patients. Whole cell recordings show the majority of cells display distinct electrophysiologic signatures and basolateral K^+^ currents consistent with type I or type II hair cell phenotypes as previously described in other species. Pharmacological approaches were used to further probe hair cell K^+^ conductances. Elucidating the properties of human vestibular hair cells paves the way to better understand and ultimately treat human vestibular disorders.

## 2 Materials and methods

### 2.1 Obtaining human tissue

The research protocol (#19-1340) was approved by the Colorado Multiple Institutional Review Board in order to obtain human inner ear tissues for the study and collect patient data via chart review. Biosafety approval was obtained for all members of the participating research team. Written, informed consent was obtained from patients undergoing translabyrinthine approaches at the University of Colorado Hospital. No compensation was provided for participating in the study. Patient charts were reviewed for demographic data, comorbidities, temporal bone imaging findings, and audiometric and vestibular testing. During the surgery cristae were obtained from the affected ear of each subject. An edited recording of the vestibular organ harvest is available online (24). Harvested organs were placed on a sheet of sterile Telfa non adherent pad which was immersed in saline (sterile isotonic 0.9% sodium chloride) in a sterile specimen container. Specimens were subsequently placed on ice and delivered to the laboratory. Vestibular tissue was typically received in the research laboratory within 30 min of explantation.

### 2.2 Cell isolation and electrophysiology

Vestibular hair cells were dissociated from human cristae for electrophysiological recordings using techniques similar to those previously utilized by our lab in gerbil and rat vestibular epithelia (25). Ampullae were extracted in saline from the specimen container and individual cristae were trimmed with care away from the membranous canal and non-sensory regions of the ampulla with micro scissors. Neuroepithelia were incubated in pH-balanced (7.4) Leibovitz’s L-15 medium with bovine serum albumin (BSA, 0.5 mg/mL) for ≥30 min at room temperature (21–24°C). Most tissue was processed immediately after it was received but in two cases individual cristae were maintained in L-15/BSA solution at 4°C overnight. Cristae were transferred to pH-balanced L-15 medium and mechanically treated in specialized recording dishes by lightly brushing a fine probe across the neuroepithelial surface several times under a dissecting microscope to dislodge hair cells. As a result, isolated hair cells or small clumps of cells settled on to the coverslip base of the recording dish and were viewed on an upright microscope (Olympus BX50 or BX51, Tokyo, Japan) using DIC optics and X40 or X60 water immersion objectives. Hair cells were considered healthy if membranes appeared relatively smooth and nuclei and excessive blebbing were not visible. Whole cell recordings were obtained from isolated hair cells up to ~5 h following dissociation.

Capillary glass (PG165T, Warner Instrument Corp., Hamden CT) was used to pull patch pipettes on a horizontal micropipette puller (Sutter Instruments P97, Novato, CA). The pipette tips were polished by brief heat application (Narishige Microforge 83, Amityville, NY, United States) and shanks were coated in Sylgard (Dow Corning, Midland, MI, United States). Whole cell tight-seal patch-clamp experiments were performed at room temperature in L-15 media with a patch pipette solution of (in mM): 115 KF, 10 KCl, 2 NaCl, 10 HEPES, 3 D-glucose, 2 MgCl_2_, and 10 EGTA, pH 7.4 with KOH (~20 mM). Electrode resistance in the bath solution ranged from 1.3 to 5.5 MΩ. Gigaohm seals were obtained on hair cell basolateral membranes and following membrane breakthrough cells were held in voltage clamp and voltage protocols applied. Membrane currents were amplified (Axopatch 1D or Axopatch 200B, Axon Instruments, Union City, CA, United States), filtered and data were acquired and analyzed using pClamp software (Axon Instruments, Union City, CA, United States) on a connected PC. Resting membrane potentials were measured by placing the amplifier in current clamp mode. Uncompensated series resistance typically ranged from 2.5–14.0 MΩ and whole cell capacitance was estimated with the patch amplifier’s capacitance compensation circuitry. Correction for liquid junction potential was applied during analysis. 4-aminopyridine (4-AP, Sigma-Aldrich, 1 mM) and 8-bromoguanosine 3′5′-cyclic monophosphate (8-Br-cGMP, Sigma-Aldrich, 0.5 mM) were dissolved in the external L-15 solution on the day of experiment and the solution pH adjusted to 7.4. 8-Br cGMP and 4-AP were perfused into the recording chamber with the use of a peristaltic pump (Gilson, flow rate 0.5–1.0 mL/min).

A standard voltage protocol was used to record hair cell ionic currents. Each cell was held at -80 mV, stepped briefly to −130 mV and then stepped to a series of potentials from −90 mV in 5 or 10 mV accretions before returning to the holding potential. Activation protocols were employed to assess voltage dependence of hair cell outward currents and data were fit with a Boltzmann function of the form:


(1) $$I/I_{\text{max}}=1/1+\exp\;\left[\left(V_{1/2}-V\right)/S\right]$$

Where V is the voltage conditioning step, V_1/2_ is half-maximum activation potential and S the slope factor.

Data were analyzed offline using p-Clamp v10 (Axon Instruments, Union City, CA, United States) and Sigmaplot (Systat Software, Palo Alto, CA). Statistical significance was determined using *t* tests for normally distributed data or the Mann–Whitney Rank Sum Test for data that failed the normality test. Data are expressed as mean ± standard deviation (S.D.) or as medians.

## 3 Results

### 3.1 Patient demographics

Sixteen consenting subjects were included in this study (9 male, 7 female). Ages ranged from 24 to 77 years with a median age of 53 years. All patients identified as white or Caucasian. One patient identified as Hispanic/Latino. All but one underwent surgery for a unilateral vestibular schwannoma and the additional patient underwent surgery for a cholesterol granuloma. The Koos score for vestibular schwannoma patients had an average value of 3.1 ± 0.96 (*n* = 15). Four patients reported dizziness, one of which described vertigo. Two patients underwent preoperative vestibular testing, both of whom exhibited asymmetric deficits on the side of the tumor. Comorbidities included obesity in four patients, hypertension in four patients, hyperlipidemia in two patients, hypothyroidism in one, and renal failure in one. Four patients were former smokers, and one was an active smoker. All patients underwent a translabyrinthine approach to the cerebellopontine angle for tumor resection.

### 3.2 Morphology of isolated hair cells

**Figure 1:**
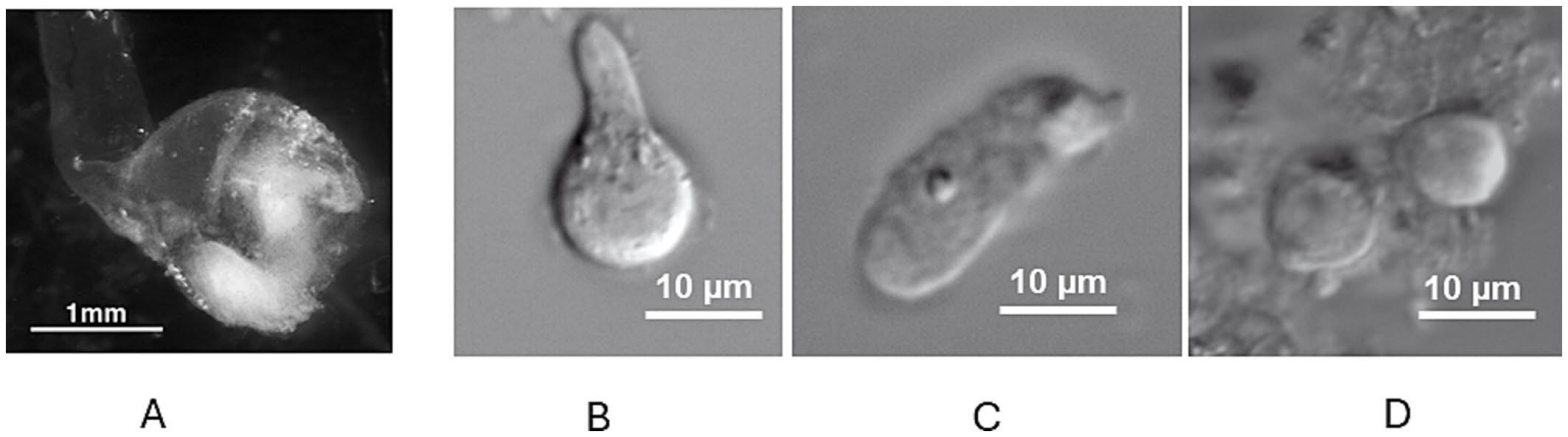
**(A)** Human ampulla with attached canal duct under low power magnification. The sensory epithelium (crista, lower right) is seen as a white structure within the translucent ampulla. **(B)** Dissociated type I hair cell with broad base and narrow neck region (cuticular plate not visible) under high power magnification. **(C)** Dissociated cylinder-shaped cell characteristic of type II hair cell. **(D)** Two adjacent spherical shaped hair cells of indeterminate type. Scale bars as indicated.

Vestibular epithelia were collected from vestibular therapeutic surgeries by the surgical team and rapidly transported to the laboratory. Ampullae containing cristae were placed in L-15 solution, examined under a dissecting microscope and evaluated. In many cases, the canal wall remained attached to the ampulla (Figure 1A). Following the isolation protocol, dissociated human vestibular hair cells appeared subjectively larger than those obtained from rodent cristae. In accordance with this observation, the mean whole cell capacitance measured in human hair cells was 8.6 ± 5.1 pF (*n* = 8). This was greater than mean values reported for hair cells from rodent crista and utricle, which are reported to range from approximately 3 to 5 pF (11, 14, 15, 23). Most dissociated cells lacked intact hair bundles (Figures 1B–D) but stereocilia were occasionally observed at the cell apices. Electron and confocal microscopy reports from human vestibular schwannoma tissue have also noted many hair cells with absent hair bundles (6, 7). A variety of cell morphologies was documented as reported previously for human hair cells dissociated using enzymatic papain and mechanical isolation (4). Some hair cells had a clear constricted neck region and a broad spherical base consistent with type I hair cell morphometric characteristics (Figure 1B). Other isolated hair cells lacked a distinct neck region and were roughly cylindrical resembling type II hair cells (Figure 1C). Another group of cells were more spheroid in appearance and based on their morphology could not be clearly categorized as type I or type II (Figure 1D). Similar short, rounded hair cells were described in intact utricles from vestibular schwannoma patients (7).

### 3.3 Electrophysiological properties of isolated vestibular hair cells

**Figure 2:**
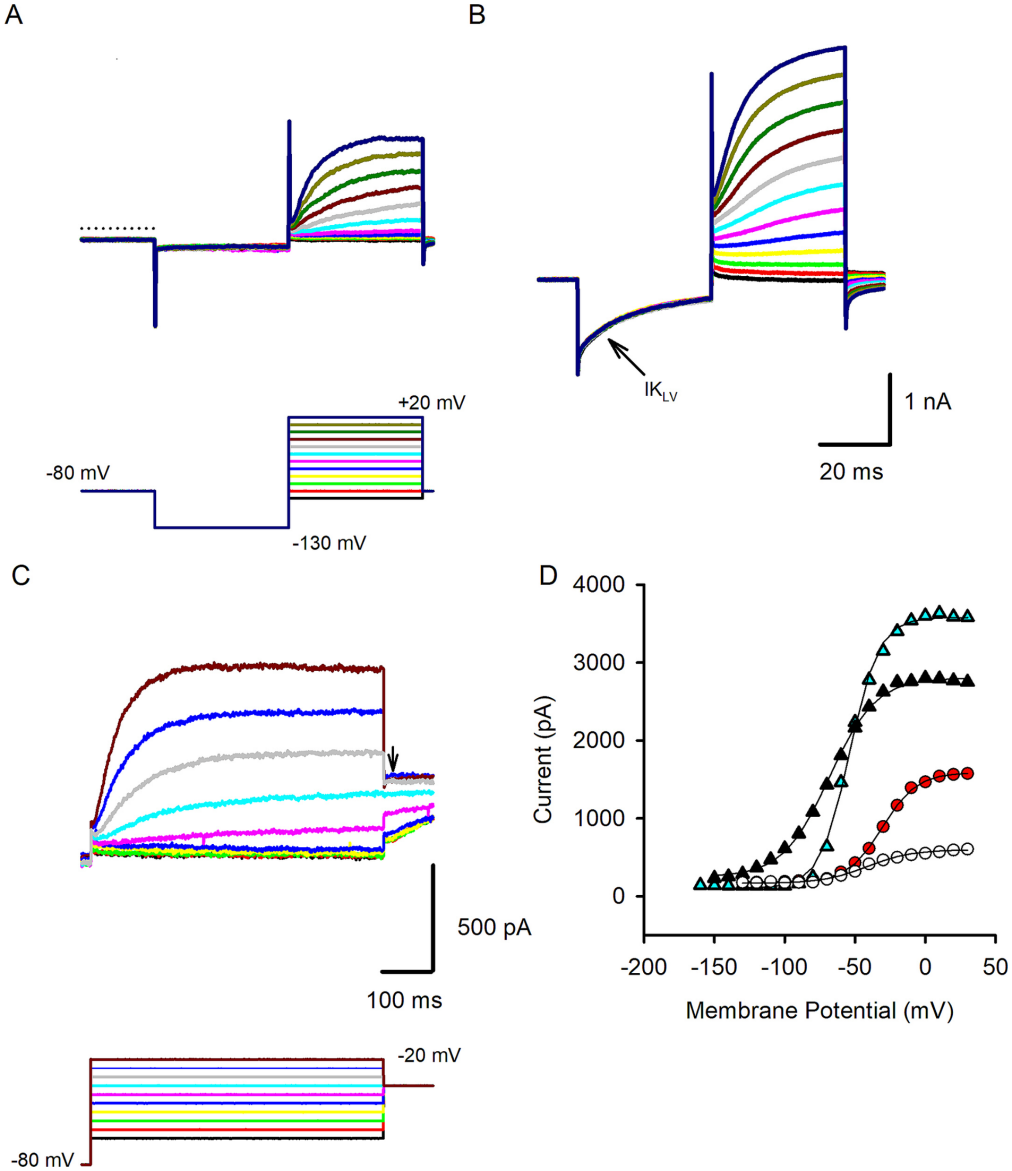
Voltage-dependent outward currents in isolated human hair cells. **(A)** A representative type II hair cell showing outward currents (upper panel) in response to the standard voltage protocol (lower panel). The membrane was held at −80 mV stepped to −130 mV and stepped in 10 mV increments between −90 and + 20 mV. Slowly developing outward currents were present at voltage steps above −50 mV. Dashed line indicates zero current level (71 year old female). **(B)** In response to the same voltage protocol an example type I hair cell showed an active resting current that partly turned off (deactivated) on stepping to −130 mV indicating the presence of I_KLV_ (arrow). At depolarized potentials large outward currents were observed that continued to increase in size over the duration of the voltage steps (56 year old male). **(C)** Activation protocol for another type II hair cell. Cell was held at −80 mV and stepped in 5 mV increments to a series of potentials before stepping to a test potential at −35 mV (arrow). Outward currents showed a small time-dependent decline at the most depolarized step (74 year old female). **(D)** Activation curves in 4 hair cells obtained from activation protocols. Currents were measured at the test step and plotted versus voltage for 2 type I hair cells (triangles) and 2 type II hair cells (circles). Plots were fit with a Boltzmann function (Equation 1, solid lines) to obtain V_1/2_ values which were − 58. 3 mV and − 43.9 mV for the 2 type I hair cells, −20.5 mV and − 31.4 mV for the 2 type II hair cells.

Following membrane breakthrough cells were held in voltage clamp, stepped to −130 mV and then stepped through a series of potentials to obtain membrane currents in whole cell recordings. Isolated hair cells demonstrated outward currents with variable properties. In a previous study, hair cells isolated from human semicircular canal and otolith organs demonstrated currents with reversal potentials close to the K^+^ equilibrium potential (between −71 and − 74 mV) indicating currents flow through K^+^ selective ion channels (3). Figure 2A shows an example of a presumed type II cell that showed little current at the holding potential but developed outward currents at steps to potentials above −50 mV. Currents were similar to delayed rectifier outward K^+^ currents described previously in mature type II rodent hair cells (14). Other isolated hair cells, some with type I-like morphology, had markedly different properties and showed low-voltage activated currents that were active around the holding potential as shown in Figure 2B. These cells demonstrated pronounced deactivation of the resting current when stepped to hyperpolarized potentials from the holding potential. Activation of large outward currents was evident at potentials depolarized above −70 mV (Figure 2B). These features are consistent with the low-voltage-activated K^+^ current (named I_K,L_ or I_KLV_) that is a striking feature of type I hair cells across vestibular end organs in rodents, birds and reptiles [reviewed in Meredith and Rennie (10)]. Here we classified cells with low-voltage-activated current as type I hair cells, although in the absence of calyx terminals to define type I hair cells we cannot be sure that all cells fell into this category. I_KLV_ bestows large resting conductance and we compared input resistance in type I hair cells with type II hair cells. Median input resistance in type I cells was 94.8 MΩ (*n* = 19), significantly lower than in type II hair cells (median 335.7 MΩ, *n* = 15, Table 1), but similar to the value of 158 MΩ reported for human type I hair cells at 15–18 weeks gestation (1). We measured resting membrane potentials in current clamp; type I cell resting potentials had an average of −62.6 ± 8.2 mV, *n* = 14 (mean ± SD) whereas mean type II hair cell resting potentials was −55.0 ± 10.1 mV, *n* = 11 (statistical significance not detected). Mean peak outward current obtained during a step to 0 mV was ~3.5 nA in type I hair cells which was more than twice the size of peak current in type II hair cells (Table 1). In response to longer duration voltage steps outward currents often showed a small degree of inactivation as shown for the type II hair cell in Figure 2C. At a voltage step to +20 mV inactivation of the outward current did not exceed 10%. The voltage-dependent activation properties of hair cell outward currents were obtained from several cells using protocols where the membrane potential was stepped to a range of voltages before the test step to evoke tail currents (Figure 2C). Activation plots for four hair cells are shown in Figure 2D. The average half-activation voltage (V_1/2_) for type I hair cells was −60.9 ± 10.8 mV, slope 9.0 ± 4.6 (*n* = 5), which is more negative than a value of −47.4 mV previously reported in a putative fetal type I cell (1). For cells that were identified as type II hair cells (including four cells with spheroid morphology), mean V_1/2_ was −36.7 ± 8.7 mV (*n* = 7), which is ~8 mV more hyperpolarized than the mean value reported for delayed rectifier K^+^ currents in type II hair cells of mouse utricle (14).

**Table 1:** Properties of isolated type I and type II human crista hair cells.

	Type I	Type II	Significance
Mean resting potential (mV)	−62.6 ± 8.2 (*n* = 14)	−55.0 ± 10.1 (*n* = 11)	*p* = 0.05
Input resistance (median, MΩ)	94.8 (*n* = 19)	335.7 (*n* = 15)	*p* < 0.001 Mann Whitney Rank Sum Test
K^+^ current, V_1/2_ (mV)	−60.9 ± 10.8 (*n* = 5)	−36.7 ± 8.7 (*n* = 7)	*p* = 0.002
K^+^ current, slope	9.0 ± 4.6 (*n* = 5)	10.5 ± 4.5 (*n* = 7)	NS (*p* = 0.574)
Peak K^+^ current amplitude at 0 mV (pA)	3,434 ± 1,274 (*n* = 15)	1,613 ± 788 (*n* = 15)	*p* < 0.001

During voltage protocols in all cells where hyperpolarizations preceded depolarizing voltage steps we saw no evidence of rapid transient inward currents preceding outward currents that would suggest the presence of Na^+^ currents. Transient Na^+^ currents are associated with immature hair cells and have been reported in human fetal vestibular hair cells (2), early postnatal rodent vestibular hair cells (25, 26), chick hair cells (27, 28) and regenerating hair cells of mouse utricle (23). However, electrophysiological expression of Na^+^ currents is lost with postnatal maturation in rodent hair cells and in this regard human hair cells that we recorded from appeared mature in their electrophysiological properties.

### 3.4 Effect of 4-aminopyridine on hair cell K^+^ currents

**Figure 3:**
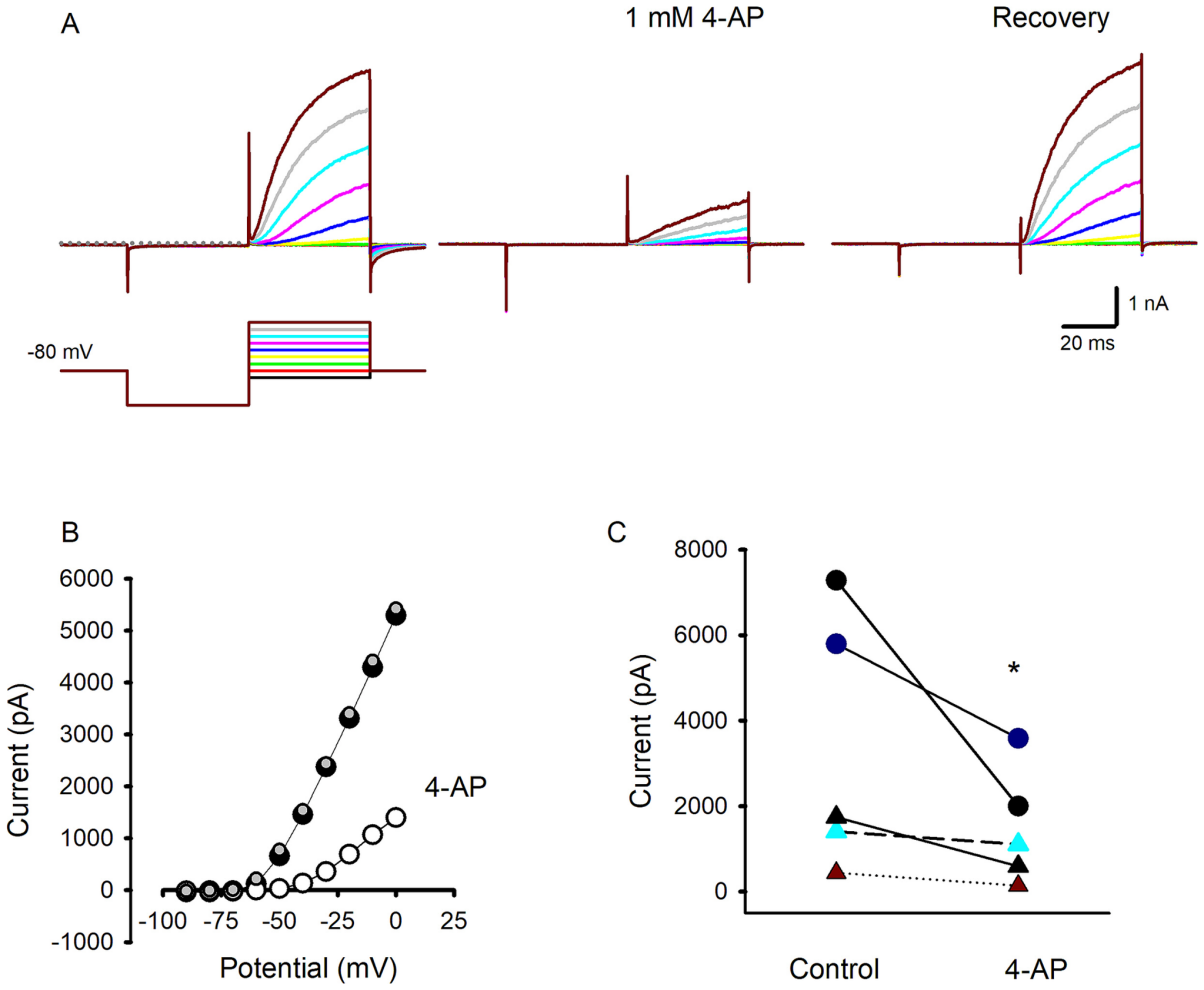
Effect of the K^+^ channel blocker 4-AP on hair cell outward currents. **(A)** Control currents in a type I hair cell in response to voltage protocol from a holding potential of −80 mV (left panel, voltage protocol shown below). A large reduction in outward currents was seen following perfusion of 1 mM 4-AP (middle panel). Recovery of currents on return to perfusion with control solution (right panel) (46 year old male). **(B)** Current–voltage relation showing peak currents in control condition (black symbols), during perfusion with 1 mM 4-AP (open symbols) and complete recovery following return to normal extracellular solution (grey symbols). **(C)** Effect of 4-AP on outward currents in 5 different hair cells following 4-AP exposure. Outward current was measured following the voltage step to +20 mV for 2 type I hair cells (circles) and 3 type II hair cells (triangles), * *p* = 0.03.

It has been demonstrated that despite their differences in voltage-dependent and kinetic properties, K^+^ currents from type I and type II hair cells from vestibular organs of birds, turtles and rodents are strongly reduced by the K^+^ channel blocker 4-aminopyridine (4-AP) (14, 25, 29–32). Since effects of K^+^ channel modulators on human hair cells have not been reported previously, we tested 4-AP in several isolated human hair cells to investigate the underlying K^+^ current characteristics. Figure 3 shows an example of a type I hair cell with large slowly activating outward currents at steps above −70 mV. Following extracellular perfusion of 1 mM 4-AP, currents in response to the voltage protocol were substantially reduced with much smaller and slowly developing remaining outward currents at voltage steps above −40 mV. Following a wash with the regular extracellular solution the outward current recovered to control values indicating the blocking action of 4-AP was reversible (Figure 3A). Currents under the different conditions are shown for the range of voltage steps in Figure 3B. The effect of 4-AP was tested in 5 cells (3 type II hair cells and 2 type I hair cells) as summarized in Figure 3C. On average 4-AP blocked peak outward current at a step to +20 mV by 53.1 ± 22.4% (*n* = 5) and confirmed that K^+^ current components in both type I and type II hair cells from human crista are reduced by extracellular application of 4-AP. In this regard human hair cell currents resemble 4-AP-sensitive delayed rectifier K^+^ currents in utricular and crista hair cells from other species.

### 3.5 Effect of cGMP analog on type I hair cell currents

**Figure 4:**
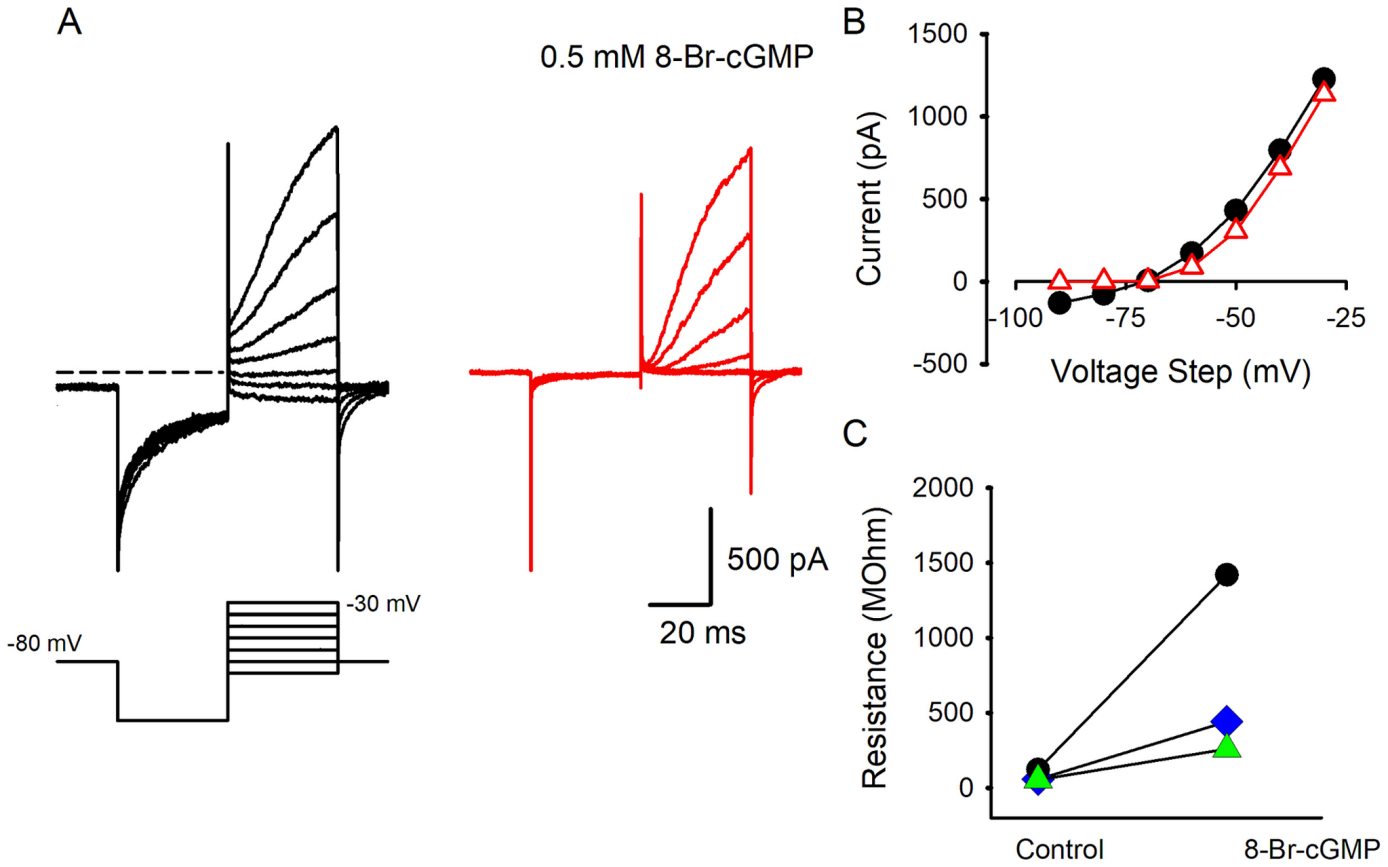
Reduction of I_KLV_ in type I hair cells by cGMP analog. **(A)** Type I hair cell recording showing control currents in response to a series of voltage steps between −90 and − 30 mV following a hyperpolarization step to −130 mV. Control currents deactivated in response to the hyperpolarization step (left panel). Following perfusion with 0.5 mM 8-Br-cGMP, deactivation of currents at the hyperpolarization step is no longer seen and smaller outward currents occur with depolarizing steps above −70 mV (right panel). **(B)** I-V plots for control currents (black circles) and in the presence of 8-Br-cGCMP (red triangles). Currents were measured at the end of each voltage step (46 year old male). **(C)** Data from 3 type I hair cells demonstrating increase in input resistance in the presence of 8-Br-cGCMP.

The Shaker K^+^ channel subunit Kv1.8 has recently been identified as a major contributor to the distinctive low voltage activated K^+^ current in type I hair cells of the mouse utricle (11). Kv1.8 channels are known to have a cyclic nucleotide binding domain (33) and in rodent type I hair cells cGMP analogs have been shown to modulate I_KLV_. Nitric oxide (NO) is a gaseous neurotransmitter that may modulate hair cell systems via cGMP. A previous report indicated that Ca^2+^ and K^+^ conductances in type II hair cells isolated from bullfrog saccule were reduced and enhanced, respectively, by gaseous NO (34). Additionally, I_KLV_ in rodent type I hair cells was shown to be modulated through a NO mechanism whereby NO producing agents and cyclic GMP (cGMP) analogs inhibited I_K,L_ in type I hair cells of rat crista (35, 36). To test whether human hair cell currents were modulated in a similar way we recorded currents from type I hair cells in the presence of 8-Bromo-cGMP (8-Br-cGMP), a nonhydrolyzable and membrane permeable form of cGMP. The effect of 8-Br-cGMP on type I hair cell K^+^ currents is shown in Figure 4, where extracellular perfusion of solution containing 0.5 mM 8 Br cGMP strongly inhibited the resting K^+^ current. Strikingly, the deactivation of I_KLV_ was suppressed following the voltage steps from −80 to −130 mV and K^+^ currents were reduced around the holding potential (Figure 4). A similar reduction in current around the holding potential was seen in 2 additional type I hair cells and as a result membrane resistance increased dramatically in the presence of solution containing 0.5 mM 8-Br-cGMP. Mean input resistance in control solution was 77.6 ± 38.5 MΩ which increased to 707.1 ± 624.1 MΩ (*n* = 3), in the presence of 8-Br-cGMP.

### 3.6 A sub-population of human hair cells exhibit hyperpolarization-activated current

**Figure 5:**
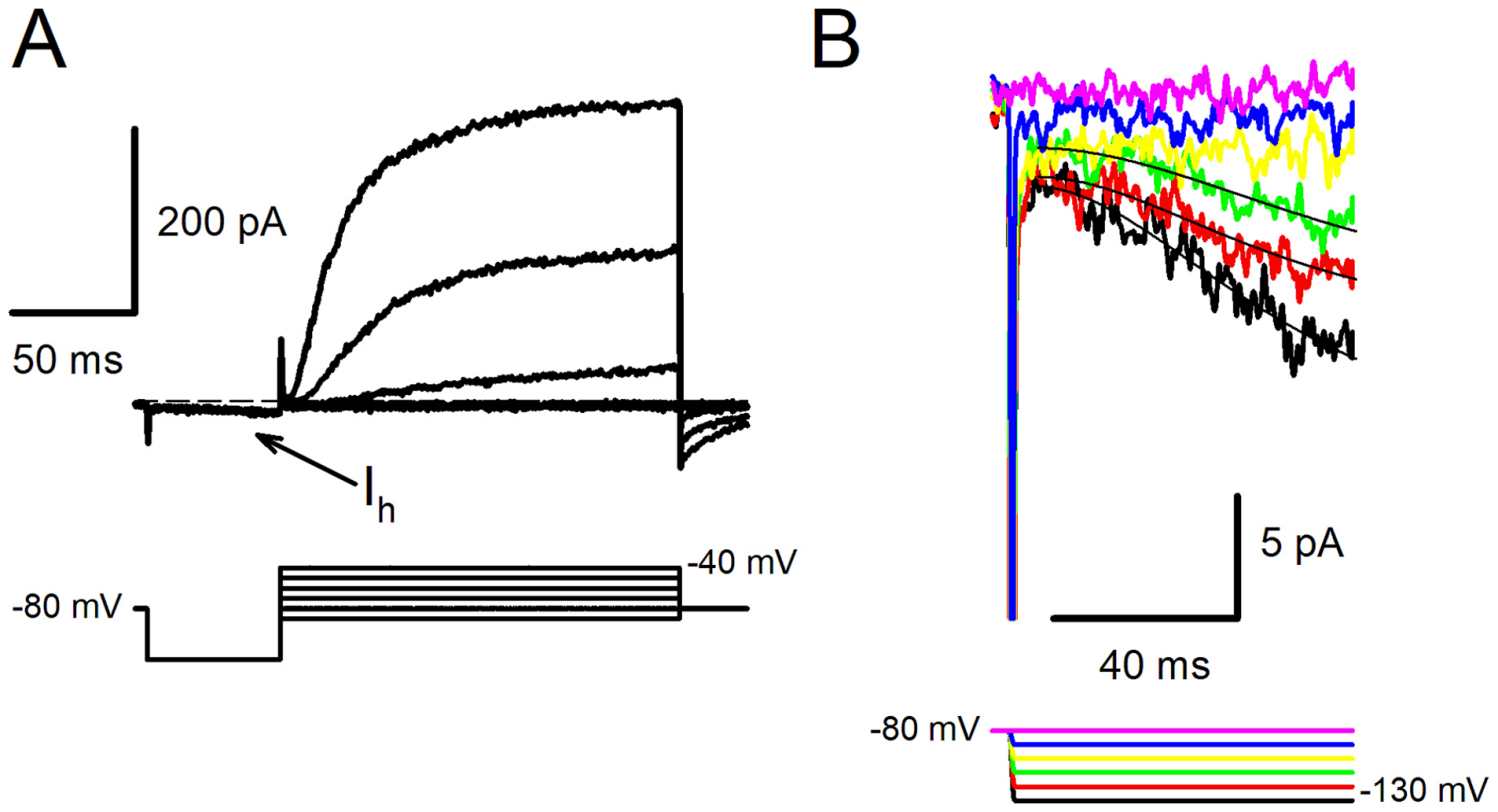
Hyperpolarization-activated currents in human hair cells. **(A)** Slowly activating inward current (I_h_) was observed during the hyperpolarization step to −130 mV (upper panel, arrow) in a type II hair cell (57 year old male). Following the 50 ms hyperpolarizing step, the membrane potential was stepped in 10 mV increments from −90 to −40 mV, outward K^+^ currents were present at −60, −50 and − 40 mV. Voltage protocol is shown in the lower panel, cell resting potential −62 mV (dashed line). **(B)** Slowly activating inward currents were evoked in the same cell in response to long duration hyperpolarizing steps between −130 and − 90 mV. Currents at the 3 most negative steps were fit with single exponential functions (superimposed black lines). Values for time constants were 35.1 ms (−130 mV), 34.2 ms (−120 mV) and 44.2 ms (−110 mV).

Figure 5A shows an example of a type II hair cell in response to a voltage protocol for steps between −80 mV and − 40 mV following a 50 ms prepulse to −130 mV. Outward K^+^ currents were preceded by small and slowly activating inward currents at the step to −130 mV (arrow). The hyperpolarization-activated current was investigated further in response to long duration (up to 1 s) voltage steps to hyperpolarized potentials more negative than −100 mV. The slowly activating currents were largest at the most negative step (Figure 5B). Slow inward currents activated by long hyperpolarizations were observed in 2 type I hair cells and 3 type II hair cells. Although small in magnitude (mean peak inward current was 17.1 ± 10.1 pA, *n* = 5), current kinetics closely resembled the hyperpolarization-activated inward current (I_h_) which has been described in vestibular hair cells of the mouse and rat utricle (13, 14, 23, 37) and gerbil crista (38). The activation of each hyperpolarization-activated current at steps to −130 mV, −120 mV and − 110 mV was fit with a single exponential and yielded time constant values of approximately 40 ms (Figure 5B). The characteristics of the slow inward currents observed in human hair cells are therefore consistent with I_h_ which is mediated by hyperpolarization-activated cyclic nucleotide-gated (HCN) channels.

## 4 Discussion

In this study, we collected vestibular epithelia from patients who were, with one exception, undergoing translabyrinthine approaches for vestibular schwannoma resection. Vestibular tissue was rapidly transported from the operating room to the laboratory where cristae were mechanically processed in physiological solutions to dissociate hair cells. We obtained isolated hair cells in short term culture that remained viable over several hours and were amenable to electrophysiological patch clamp recordings. We obtained multiple recordings from hair cells isolated from adult human cristae across a wide range of ages and performed pharmacological experiments to examine underlying properties of voltage and cyclic-nucleotide gated ion channels. Although important groundwork for hair cell physiology has been performed in animal studies, elucidating human hair cell properties is essential for interpretation of clinical data. The use of surgically removed tissue that would otherwise be discarded is an excellent source for translational approaches.

### 4.1 Human vestibular hair cells exhibit diverse K^+^ currents

We found that the electrophysiological membrane properties of isolated human hair cells were dominated by voltage dependent K^+^ conductances and were broadly consistent with mature type I and type II categories as previously described in other vertebrate species (14, 15, 29, 32, 37, 39, 40). Type I hair cells showed low voltage-activated current around their mean resting potential of −63 mV and large outward K^+^ currents with depolarizations. Type II hair cells demonstrated smaller outward K^+^ currents which activated with membrane depolarizations close to the mean resting membrane potential of −55 mV. Half-activation voltage values for K^+^ currents were significantly more negative in type I hair cells resulting in low input resistance values. Previous V_1/2_ values were reported to range from −55 to −88 mV in type I hair cells from rodent, turtle and pigeon vestibular epithelia (29, 30, 32) and similarly ~50% of current was activated at rest in human type I hair cells. Because of this large resting conductance, type I hair cells and their calyx afferents are inherently capable of responding rapidly and linearly to mechanosensory signals (41).

We demonstrated that I_KLV_ in human type I hair cells was blocked by 4-AP, as described for I_KLV_ in type I hair cells from other species including birds, reptiles and small mammals (29–32, 40). A residual outward current remained in hair cells following 4-AP application which activated slowly over time at depolarized potentials above rest. Candidates for ion channel subunits mediating type I hair cell delayed rectifier K^+^ currents include Kv1, Kv7 and erg channels (11, 42–44). Recent evidence provides strong support for Kv1.8 channels, since Kv1.8 knockout mice demonstrated hearing and balance deficits (45) and their type I hair cells lack electrophysiological expression of low-voltage activated K^+^ conductance (11). Further, human KCNA10 (Kv1.8) currents were substantially blocked by 2 mM 4-AP (> 60%) and slightly reduced by 8-Br-cGMP (33) when expressed in oocytes, properties shared with type I hair cell currents described here.

We observed that the resting K^+^ conductance in human type I hair cells decreased following exposure to the cGMP analog 8-Br cGMP. A similar modulation has been described in rodent type I hair cells. *In vivo*, intracellular cGMP levels may increase following nitric oxide (NO) release from cells such as vestibular efferent neurons within the vestibular epithelium. Calyx afferent endings surround type I hair cells and are contacted by vestibular efferent terminals which contain NO synthase in small mammals (46, 47). Released gaseous NO could enter type I hair cells via diffusion, increase intracellular cGMP levels and result in closure of I_KLV_ channels. The precise mechanism of action remains unclear, but Kv1.8 channels notably have a cyclic nucleotide binding site suggesting that cGMP could interact directly with these channels (11, 33). Alternatively increased cGMP levels may trigger intracellular cascades leading to the closure of membrane K^+^ channels (35, 36, 48). In either scenario reduction of K^+^ channel activity would increase type I hair cell membrane resistance, depolarize the resting potential and render cells more responsive to mechanosensory currents originating at the hair bundle. Our findings confirm that cGMP modulates human type I hair cell K^+^ currents and support a common vestibular efferent control mechanism across mammalian species.

A population of isolated hair cells from human crista lacked large resting K^+^ current, their input resistance was significantly higher than type I hair cells and delayed rectifier K^+^ currents were present at depolarized potentials. These properties are characteristic of type II hair cells as described in rodents (14, 40, 49) and in a few cells from mature and developing human vestibular epithelia (1, 3). Like rodent type II hair cells (14, 37, 40), a large part of the delayed rectifier K^+^ current in the human cell population was inhibited by 4-AP. Although we observed some slow inactivation of K^+^ current in human type II hair cells at depolarized potentials (< 10%), rapidly activating and rapidly inactivating “A”-type currents as described in type II hair cells of turtles, birds and mice (11, 12, 17, 29) were not seen. Rapid A currents are attributed to Kv1.4 channels in these species (50, 51). Kv1.4 protein is encoded by the KCNA4 gene but was not detected in human vestibular hair cells with transcriptomic analysis (7). Kv1.8 and Kv7 channels contribute to slower outward K^+^ currents in type II hair cells in rodents (11, 52) and transcripts for these K^+^ channels are present in human utricular hair cells (7) and likely underlie delayed rectifiers in human type II hair cells. Calcium-activated K^+^ currents were described in type II hair cells isolated from guinea pig crista (40) and although not directly investigated here might also contribute to the non-inactivating K^+^ currents observed in human hair cells.

### 4.2 Evidence for HCN channels in human hair cells

We recorded a slow hyperpolarization-activated inward current, I_h_, in a few isolated hair cells. Although we did not pharmacologically characterize this current, it shared features of the mixed cation inward rectifier current described in the majority of type I and type II vestibular hair cells of the mouse utricle at early postnatal ages (13, 14). HCN1 channels were identified as the predominant subunits mediating I_h_, since mice lacking inner ear HCN1 channels had reduced vestibular evoked potentials, showed deficiencies in balance tasks and their hair cells had absent I_h_ (13). In mature pigeon vestibular epithelia, I_h_ was present in approximately 30% of type II hair cells and was more frequently found in central epithelial areas (12). The activation time constant for hyperpolarization-activated currents of ~50 ms in rodent vestibular type II hair cells (13, 14, 38) is like the activation time course of I_h_ described here in human hair cells. A similar small inwardly rectifying current was previously described in a single type II hair cell isolated from human vestibular epithelia, although the reported time constant for that cell of 133 ms was slower (3). HCN channels are associated with the large synaptic appositions between type I hair cells and their calyx neurons and can speed up postsynaptic potentials associated with quantal transmission (38, 53). HCN channels are also important for an uncustomary form of synaptic communication termed non-quantal transmission (54–56). Non-quantal mediated signals enable highly synchronized and extremely short latency responses in vestibular afferents in guinea pig utricles (57). Non-quantal transmission mechanisms in human vestibular neuroepithelia remain to be demonstrated but are hypothesized to mediate extremely fast vestibular signals that could permit rapid bodily reflexes.

### 4.3 Implications for functionality in peripheral vestibular organs

Hair cell counts in rodent vestibular epithelia revealed that there are roughly equal numbers of type I and type II hair cells where the presence of calyx terminals was used to define type I hair cells (58, 59). Slightly greater numbers of type I hair cells relative to type II hair cells were reported in squirrel monkey crista and human utricles (60, 61). Age, genetic factors, trauma and ototoxic compounds can contribute to hair cell loss and result in vestibular dysfunction. Sporadic vestibular schwannomas are typically slow growing and associated with hearing loss and vestibular deficits in patients. A marked loss of hair cells has been described in epithelia from vestibular schwannoma patients and remaining hair cells often had stereociliary abnormalities (6, 7). Factors released from vestibular schwannomas *in vitro* resulted in damage and loss of auditory hair cells in cochlear explants (62) and might also cause hair cell loss in vestibular organs. Recent studies suggest that adult human utricles retain a capacity for hair cell regeneration (5, 6). Transcriptomes associated with hair cell precursors were increased in utricles from vestibular schwannoma patients compared to those from organ donors with normal non-diseased ears (7). How pathophysiological changes associated with vestibular schwannomas impact the overall electrical activity in hair cells and their associated afferent neurons remains unresolved, but our results indicate that remaining type I and type II hair cells maintain typical biophysical characteristics and basolateral conductances in crista extracted from schwannoma patients.

The distinct ionic conductances of type I and type II hair cell types emerge during early postnatal development in rodents. Type I hair cells start to display the characteristic low voltage-activated K^+^ current during the first few days after birth and it is present in all type I hair cells by the end of the first month (14, 25, 44, 63). Developing hair cells in human semi-intact cristae were reported to show small K^+^ conductances characteristic of immature vestibular hair cells in other species and the presence of I_KLV_ was first noted in some fetal hair cells at 15 weeks gestation (1). Developing rodent vestibular hair cells express Na^+^ currents over a brief time window but their electrophysiological expression is lost with maturation (14, 25, 26, 63). Transient inward Na^+^ currents with partial sensitivity to tetrodotoxin were also reported in developing human fetal hair cells representing an immature phenotype (1, 2). In adult mouse utricle where hair cells were ablated, regenerated hair cells also showed electrophysiological properties consistent with immature hair cells. Regenerated hair cells exhibited small mechanotransduction currents, outward K^+^ currents and transient Na^+^ currents but lacked hyperpolarization-activated currents (23). Given recent findings supporting the capacity for hair cell regeneration in utricles surgically removed from patients with vestibular schwannomas, hair cells with immature basolateral conductances might be expected. However, we did not find evidence of Na^+^ currents in any hair cells in our samples and properties were consistent with type I and type II electrophysiological properties supporting a mature functional segregation. An improved understanding of how hair cells are lost and could potentially be replaced by precursors in human vestibular epithelia is needed. Elucidating how electrophysiological phenotypes relate to vestibular function is also required for therapies directed towards functional restoration.

## Data Availability

The raw data supporting the conclusions of this article will be made available by the authors, without undue reservation.

## References

[1] Lim R Drury HR Camp AJ Tadros MA Callister RJ Brichta AM. Preliminary characterization of voltage-activated whole-cell currents in developing human vestibular hair cells and calyx afferent terminals. J Assoc Res Otolaryngol. (2014) 15:755–66. doi: 10.1007/s10162-014-0471-y, PMID: 24942706 PMC4164689

[2] Quinn RK Drury HR Cresswell ET Tadros MA Nayagam BA Callister RJ. Expression and physiology of voltage-gated sodium channels in developing human inner ear. Front Neurosci. (2021) 15:733291. doi: 10.3389/fnins.2021.733291, PMID: 34759790 PMC8575412

[3] Oghalai JS Holt JR Nakagawa T Jung TM Coker NJ Jenkins HA. Ionic currents and electromotility in inner ear hair cells from humans. J Neurophysiol. (1998) 79:2235–9. doi: 10.1152/jn.1998.79.4.2235, PMID: 9535985

[4] Oghalai JS Nakagawa T Jenkins HA Jung TM Eatock RA Holt JR. Harvesting human hair cells. Ann Otol Rhinol Laryngol. (2000) 109:9–16. doi: 10.1177/000348940010900102, PMID: 10651405

[5] Taylor RR Filia A Paredes U Asai Y Holt JR Lovett M. Regenerating hair cells in vestibular sensory epithelia from humans. eLife. (2018) 7:e34817. doi: 10.7554/eLife.34817, PMID: 30019672 PMC6078492

[6] Taylor RR Jagger DJ Saeed SR Axon P Donnelly N Tysome J. Characterizing human vestibular sensory epithelia for experimental studies: new hair bundles on old tissue and implications for therapeutic interventions in ageing. Neurobiol Aging. (2015) 36:2068–84. doi: 10.1016/j.neurobiolaging.2015.02.013, PMID: 25818177 PMC4436436

[7] Wang T Ling AH Billings SE Hosseini DK Vaisbuch Y Kim GS. Single-cell transcriptomic atlas reveals increased regeneration in diseased human inner ear balance organs. Nat Commun. (2024) 15:4833. doi: 10.1038/s41467-024-48491-y, PMID: 38844821 PMC11156867

[8] Wersall J. Studies on the structure and innervation of the sensory epithelium of the cristae ampulares in the guinea pig; a light and electron microscopic investigation. Acta Otolaryngol Suppl. (1956) 126:1–85. PMID: 13326368

[9] Eatock RA Songer JE. Vestibular hair cells and afferents: two channels for head motion signals. Annu Rev Neurosci. (2011) 34:501–34. doi: 10.1146/annurev-neuro-061010-113710, PMID: 21469959

[10] Meredith FL Rennie KJ. Channeling your inner ear potassium: K+ channels in vestibular hair cells. Hear Res. (2016) 338:40–51. doi: 10.1016/j.heares.2016.01.015, PMID: 26836968

[11] Martin HR Lysakowski A Eatock RA. The potassium channel subunit Kv1.8 (Kcna10) is essential for the distinctive outwardly rectifying conductances of type I and II vestibular hair cells. eLife. (2024) 13:RP94342. doi: 10.7554/eLife.94342.1 39625061 PMC11614384

[12] Weng T Correia MJ. Regional distribution of ionic currents and membrane voltage responses of type II hair cells in the vestibular neuroepithelium. J Neurophysiol. (1999) 82:2451–61. doi: 10.1152/jn.1999.82.5.2451, PMID: 10561418

[13] Horwitz GC Risner-Janiczek JR Jones SM Holt JR. Hcn channels expressed in the inner ear are necessary for normal balance function. J Neurosci. (2011) 31:16814–25. doi: 10.1523/JNEUROSCI.3064-11.2011, PMID: 22090507 PMC3477615

[14] Rüsch A Lysakowski A Eatock RA. Postnatal development of type I and type II hair cells in the mouse utricle: Acquisition of Voltage-Gated Conductances and Differentiated Morphology. J Neurosci. (1998) 18:7487–501. doi: 10.1523/JNEUROSCI.18-18-07487.1998, PMID: 9736667 PMC6793223

[15] Rennie KJ Ricci AJ Correia MJ. Electrical filtering in gerbil isolated type I semicircular canal hair cells. J Neurophysiol. (1996) 75:2117–23. doi: 10.1152/jn.1996.75.5.2117, PMID: 8734607

[16] Correia MJ Rennie KJ Koo P. Return of potassium ion channels in regenerated hair cells: possible pathways and the role of intracellular calcium signaling. Ann N Y Acad Sci. (2001) 942:228–40. doi: 10.1111/j.1749-6632.2001.tb03749.x, PMID: 11710465

[17] Masetto S Correia MJ. Electrophysiological properties of vestibular sensory and supporting cells in the labyrinth slice before and during regeneration. J Neurophysiol. (1997) 78:1913–27. doi: 10.1152/jn.1997.78.4.1913, PMID: 9325360

[18] Masetto S Correia MJ. Ionic currents in regenerating avian vestibular hair cells. Int J Dev Neurosci. (1997) 15:387–99. doi: 10.1016/S0736-5748(96)00099-8, PMID: 9263021

[19] Rubel EW Furrer SA Stone JS. A brief history of hair cell regeneration research and speculations on the future. Hear Res. (2013) 297:42–51. doi: 10.1016/j.heares.2012.12.014, PMID: 23321648 PMC3657556

[20] Golub JS Tong L Ngyuen TB Hume CR Palmiter RD Rubel EW. Hair cell replacement in adult mouse utricles after targeted ablation of hair cells with diphtheria toxin. J Neurosci. (2012) 32:15093–105. doi: 10.1523/JNEUROSCI.1709-12.2012, PMID: 23100430 PMC3544304

[21] Sayyid ZN Wang T Chen L Jones SM Cheng AG. Atoh1 directs regeneration and functional recovery of the mature mouse vestibular system. Cell Rep. (2019) 28:312–324.e4. doi: 10.1016/j.celrep.2019.06.028, PMID: 31291569 PMC6659123

[22] Staecker H Praetorius M Baker K Brough DE. Vestibular hair cell regeneration and restoration of balance function induced by math1 gene transfer. Otol Neurotol. (2007) 28:223–31. doi: 10.1097/MAO.0b013e31802b3225, PMID: 17255891

[23] González-Garrido A Pujol R López-Ramírez O Finkbeiner C Eatock RA Stone JS. The differentiation status of hair cells that regenerate naturally in the vestibular inner ear of the adult mouse. J Neurosci. (2021) 41:7779–96. doi: 10.1523/JNEUROSCI.3127-20.2021, PMID: 34301830 PMC8445055

[24] OTOlivia. Harvesting vestibular organs – translabyrinthine approach YouTube (2023). Available at: https://www.youtube.com/watch?v=BxdAUt8zjHg&rco=1.

[25] Li GQ Meredith FL Rennie KJ. Development of K+ and Na+ conductances in rodent postnatal semicircular canal type I hair cells. Am J Phys Regul Integr Comp Phys. (2010) 298:R351–8. doi: 10.1152/ajpregu.00460.2009, PMID: 19939976 PMC2828173

[26] Wooltorton JR Gaboyard S Hurley KM Price SD Garcia JL Zhong M. Developmental changes in two voltage-dependent sodium currents in utricular hair cells. J Neurophysiol. (2007) 97:1684–704. doi: 10.1152/jn.00649.2006, PMID: 17065252

[27] Masetto S Bosica M Correia MJ Ottersen OP Zucca G Perin P. Na+ currents in vestibular type I and type II hair cells of the embryo and adult chicken. J Neurophysiol. (2003) 90:1266–78. doi: 10.1152/jn.01157.2002, PMID: 12702715

[28] Sokolowski BH Stahl LM Fuchs PA. Morphological and physiological development of vestibular hair cells in the organ-cultured otocyst of the chick. Dev Biol. (1993) 155:134–46. doi: 10.1006/dbio.1993.1013, PMID: 8416829

[29] Brichta AM Aubert A Eatock RA Goldberg JM. Regional analysis of whole cell currents from hair cells of the turtle posterior crista. J Neurophysiol. (2002) 88:3259–78. doi: 10.1152/jn.00770.2001, PMID: 12466445

[30] Rennie KJ Correia MJ. Potassium currents in mammalian and avian isolated type I semicircular canal hair cells. J Neurophysiol. (1994) 71:317–29. doi: 10.1152/jn.1994.71.1.317, PMID: 8158233

[31] Ricci AJ Rennie KJ Correia MJ. The delayed rectifier, I kh, is the major conductance in type I vestibular hair cells across vestibular end organs. Pflugers Arch. (1996) 432:34–42. doi: 10.1007/s004240050102, PMID: 8662265

[32] Rusch A Eatock RA. A delayed rectifier conductance in type I hair cells of the mouse utricle. J Neurophysiol. (1996) 76:995–1004. doi: 10.1152/jn.1996.76.2.995, PMID: 8871214

[33] Lang R Lee G Liu W Tian S Rafi H Orias M. Kcna10: a novel ion channel functionally related to both voltage-gated potassium and Cng cation channels. Am J Physiol Renal Physiol. (2000) 278:F1013–21. doi: 10.1152/ajprenal.2000.278.6.F1013, PMID: 10836990

[34] Lv P Rodriguez-Contreras A Kim HJ Zhu J Wei D Choong-Ryoul S. Release and elementary mechanisms of nitric oxide in hair cells. J Neurophysiol. (2010) 103:2494–505. doi: 10.1152/jn.00017.2010, PMID: 20220083 PMC2867581

[35] Behrend O Schwark C Kunihiro T Strupp M. Cyclic Gmp inhibits and shifts the activation curve of the delayed-rectifier (I[K1]) of type I mammalian vestibular hair cells. Neuroreport. (1997) 8:2687–90. doi: 10.1097/00001756-199708180-00010, PMID: 9295101

[36] Chen JW Eatock RA. Major potassium conductance in type I hair cells from rat semicircular canals: characterization and modulation by nitric oxide. J Neurophysiol. (2000) 84:139–51. doi: 10.1152/jn.2000.84.1.139, PMID: 10899192

[37] Lennan G Steinacker A Lehouelleur J Sans A. Ionic currents and current-clamp depolarisations of type I and type II hair cells from the developing rat utricle. Pflugers Arch. (1999) 438:40–6. doi: 10.1007/s004240050877, PMID: 10370085

[38] Meredith FL Benke TA Rennie KJ. Hyperpolarization-activated current (I h) in vestibular calyx terminals: characterization and role in shaping postsynaptic events. J Assoc Res Otolaryngol. (2012) 13:745–58. doi: 10.1007/s10162-012-0342-3, PMID: 22825486 PMC3505587

[39] Correia MJ Lang DG. An electrophysiological comparison of solitary type I and type II vestibular hair cells. Neurosci Lett. (1990) 116:106–11. doi: 10.1016/0304-3940(90)90394-O, PMID: 2259440

[40] Griguer C Kros C Sans A Lehouelleur J. Potassium currents in type II vestibular hair cells isolated from the guinea-pig's crista ampullaris. Pflugers Arch. (1993) 425:344–52. doi: 10.1007/BF00374185, PMID: 8060388

[41] Eatock RA. Specializations for fast signaling in the Amniote vestibular inner ear. Integr Comp Biol. (2018) 58:341–50. doi: 10.1093/icb/icy069, PMID: 29920589 PMC6104706

[42] Holt JC Chatlani S Lysakowski A Goldberg JM. Quantal and nonquantal transmission in calyx-bearing fibers of the turtle posterior crista. J Neurophysiol. (2007) 98:1083–101. doi: 10.1152/jn.00332.2007, PMID: 17596419 PMC3397384

[43] Hotchkiss K Harvey M Pacheco M Sokolowski B. Ion channel proteins in mouse and human vestibular tissue. Otolaryngol Head Neck Surg. (2005) 132:916–23. doi: 10.1016/j.otohns.2005.01.022, PMID: 15944564

[44] Hurley KM Gaboyard S Zhong M Price SD Wooltorton JR Lysakowski A. M-like K+ currents in type I hair cells and calyx afferent endings of the developing rat utricle. J Neurosci. (2006) 26:10253–69. doi: 10.1523/JNEUROSCI.2596-06.2006, PMID: 17021181 PMC6674627

[45] Lee SI Conrad T Jones SM Lagziel A Starost MF Belyantseva IA. A null mutation of mouse Kcna10 causes significant vestibular and mild hearing dysfunction. Hear Res. (2013) 300:1–9. doi: 10.1016/j.heares.2013.02.009, PMID: 23528307 PMC3684051

[46] Hess A Bloch W Su J Stennert E Addicks K Michel O. Localisation of the nitric oxide (no)/cgmp-pathway in the vestibular system of guinea pigs. Neurosci Lett. (1998) 251:185–8. doi: 10.1016/S0304-3940(98)00532-1, PMID: 9726374

[47] Lysakowski A Singer M. Nitric oxide synthase localized in a subpopulation of vestibular efferents with Nadph diaphorase histochemistry and nitric oxide synthase immunohistochemistry. J Comp Neurol. (2000) 427:508–21. doi: 10.1002/1096-9861(20001127)427:4<508::AID-CNE2>3.0.CO;2-L, PMID: 11056461 PMC12058275

[48] Rennie KJ. Modulation of the resting potassium current in type I vestibular hair cells by cGMP. In: Berlin CI Hood LJ Ricci A, editors. Hair cell micromechanics and otoacoustic emissions. Clifton Park, NY: Singular Press (2002) 79–89.

[49] Rennie K Ashmore J. Ionic currents in isolated vestibular hair cells from the guinea-pig crista ampullaris. Hear Res. (1991) 51:279–91. doi: 10.1016/0378-5955(91)90044-A, PMID: 2032962

[50] Correia MJ Weng T Prusak D Wood TG. Kvβ1. 1 associates with Kvα1. 4 in chinese hamster ovary cells and pigeon type II vestibular hair cells and enhances the amplitude, inactivation and negatively shifts the steady-state inactivation range. Neuroscience. (2008) 152:809–20. doi: 10.1016/j.neuroscience.2008.01.021, PMID: 18313857 PMC3014264

[51] Mcinturff S Burns JC Kelley MW. Characterization of spatial and temporal development of type I and type II hair cells in the mouse utricle using new cell-type-specific markers. Biol Open. (2018) 7:bio038083. doi: 10.1242/bio.038083, PMID: 30455179 PMC6262869

[52] Rennie KJ Weng T Correia MJ. Effects of Kcnq channel blockers on K(+) currents in vestibular hair cells. Am J Physiol Cell Physiol. (2001) 280:C473–80. doi: 10.1152/ajpcell.2001.280.3.C473, PMID: 11171566

[53] Meredith FL Vu TA Gehrke B Benke TA Dondzillo A Rennie KJ. Expression of hyperpolarization-activated current (I h) in zonally defined vestibular calyx terminals of the crista. J Neurophysiol. (2023) 129:1468–81. doi: 10.1152/jn.00135.2023, PMID: 37198134 PMC10259860

[54] Contini D Price SD Art JJ. Accumulation of K+ in the synaptic cleft modulates activity by influencing both vestibular hair cell and calyx afferent in the turtle. J Physiol. (2017) 595:777–803. doi: 10.1113/JP273060, PMID: 27633787 PMC5285615

[55] Govindaraju AC Quraishi IH Lysakowski A Eatock RA Raphael RM. Nonquantal transmission at the vestibular hair cell–calyx synapse: Klv currents modulate fast electrical and slow K+ potentials. Proc Natl Acad Sci. (2023) 120:e2207466120. doi: 10.1073/pnas.2207466120, PMID: 36595693 PMC9926171

[56] Songer JE Eatock RA. Tuning and timing in mammalian type I hair cells and calyceal synapses. J Neurosci. (2013) 33:3706–24. doi: 10.1523/JNEUROSCI.4067-12.2013, PMID: 23426697 PMC3857958

[57] Pastras CJ Curthoys IS Asadnia M Mcalpine D Rabbitt RD Brown DJ. Evidence that ultrafast nonquantal transmission underlies synchronized vestibular action potential generation. J Neurosci. (2023) 43:7149–57. doi: 10.1523/JNEUROSCI.1417-23.2023, PMID: 37775302 PMC10601366

[58] Desai SS Ali H Lysakowski A. Comparative morphology of rodent vestibular periphery. II Cristae ampullares. J Neurophysiol. (2005) 93:267–80. doi: 10.1152/jn.00747.2003 15240768 PMC12513555

[59] Desai SS Zeh C Lysakowski A. Comparative morphology of rodent vestibular periphery. I. Saccular and utricular maculae. J Neurophysiol. (2005) 93:251–66. doi: 10.1152/jn.00746.2003 15240767 PMC12456082

[60] Fernandez C Lysakowski A Goldberg JM. Hair-cell counts and afferent innervation patterns in the cristae ampullares of the squirrel monkey with a comparison to the chinchilla. J Neurophysiol. (1995) 73:1253–69. doi: 10.1152/jn.1995.73.3.1253, PMID: 7608769

[61] Gopen Q Lopez I Ishiyama G Baloh RW Ishiyama A. Unbiased stereologic type I and type II hair cell counts in human utricular macula. Laryngoscope. (2003) 113:1132–8. doi: 10.1097/00005537-200307000-00007, PMID: 12838010

[62] Dilwali S Landegger LD Soares VY Deschler DG Stankovic KM. Secreted factors from human vestibular schwannomas can cause cochlear damage. Sci Rep. (2015) 5:18599. doi: 10.1038/srep18599, PMID: 26690506 PMC4686978

[63] Géléoc GS Risner JR Holt JR. Developmental acquisition of voltage-dependent conductances and sensory signaling in hair cells of the embryonic mouse inner ear. J Neurosci. (2004) 24:11148–59. doi: 10.1523/JNEUROSCI.2662-04.2004, PMID: 15590931 PMC2638092

